# Pharmacogenomics in clinical trials: an overview

**DOI:** 10.3389/fphar.2023.1247088

**Published:** 2023-10-20

**Authors:** Rita Nogueiras-Álvarez

**Affiliations:** Clinical Trials Unit, Bioaraba Health Research Institute, Vitoria-Gasteiz, Spain

**Keywords:** pharmacogenomics, pharmacogenetics, clinical trials, personalised medicine, clinical pharmacology, clinical research

## Abstract

With the trend towards promoting personalised medicine (PM), the application of pharmacogenetics and pharmacogenomics (PGx) is of growing importance. For the purposes of clinical trials, the inclusion of PGx is an additional tool that should be considered for improving our knowledge about the effectiveness and safety of new drugs. A search of available clinical trials containing pharmacogenetic and PGx information was conducted on ClinicalTrials.gov. The results show there has been an increase in the number of trials containing PGx information since the 2000 s, with particular relevance in the areas of Oncology (28.43%) and Mental Health (10.66%). Most of the clinical trials focus on treatment as their primary purpose. In those clinical trials entries where the specific genes considered for study are detailed, the most frequently explored genes are *CYP2D6* (especially in Mental Health and Pain), *CYP2C9* (in Hematology), *CYP2C19* (in Cardiology and Mental Health) and *ABCB1* and *CYP3A5* (particularly prominent in Transplantation and Cardiology), among others. Researchers and clinicans should be trained in pharmacogenetics and PGx in order to be able to make a proper interpretation of this data, contributing to better prescribing decisions and an improvement in patients’ care, which would lead to the performance of PM.

## 1 Introduction

Personalised medicine (PM) was first defined in 2015 as a medical model using characterisation of individuals’ phenotypes and genotypes (e.g., molecular profiling, medical imaging, lifestyle data) for tailoring the right therapeutic strategy for the right person at the right time, and/or to determine the predisposition to disease and/or to deliver timely and targeted prevention. PM relates to the broader concept of patient-centred care, which takes into account that, in general, healthcare systems need to better respond to patient needs (European Union, 2023).

In this context, insights from pharmacogenetics and pharmacogenomics (PGx) become extremely useful to achieve such a level of personalised therapy.

While pharmacogenetics studies the individual variations in drug response due to genetic causes (Motulsky, 1957; Evans and Ckarke, 1961; Nebert, 1999), PGx is a broader based term that encompasses the simultaneous impact of multiple mutations in the genome that may determine a person response to drugs (Dere and Suto, 2009; Adams, 2008).

Although it is noticeable that drug data sheets now contain information on pharmacogenetic and PGx recommendations, not all the regulatory agencies have implemented this type of information to the same extent. The Pharmacogenomics Knowledge Base (PharmGKB) (Pharmacogenomics Knowledge Base, 2023; Whirl-Carrillo et al., 2021; Whirl-Carrillo et al., 2012), under its “Drug Label Annotations” section provides pharmacogenetic information included in the summary of product characteristics of drugs approved by different regulatory agencies. See Supplementary Material.

There is a World Health Organization’s publication from 2007 which already pointed out that PGx would cause significant changes in pharmacological research at the level of clinical trials conduct (Boulyjenkov et al., 2007).

Although the ethical challenges associated with the use of genetic information in clinical research should not be overlooked, the inclusion of PGx testing in clinical trials can help in the development of new medicines by contributing to a better understanding of their efficacy and safety (Pandya, 2017).

In this regard, clinical trials on medicinal products for human are a cornerstone for PM (Beccia et al., 2022; European Commission Directorate-General for Research and Innovation, 2011).

## 2 Materials and methods

A search on ClinicalTrials.gov (ClinicalTrials, 2023) was performed in order to review the clinical trials including PGx information available at the database. The terms “pharmacogenetics,” “pharmacogenetics and PGx studies,” “genetic” and “single-nucleotide polymorphisms” were included in the search field and the results were limited to interventional studies in order to obtain clinical trials information only.

The clinical trials were classified according to the health area they related to and their design.

Information about the clinical trial’s location and characteristics from the enrolled participants and main genes studied were also obtained.

## 3 Results

The database search (from inception through 3 June 2023) returned 350,728 results of registered “interventional studies” (clinical trials). In the advanced search option, the “pharmacogenetics” term was added and 604 results of registered clinical trials that included “pharmacogenetics” or “pharmacogenetics and PGx studies” terms were obtained. To expand the scope and to ensure that those clinical trials that have not included those terms in their protocol description could be excluded, the terms “genetic” and “single-nucleotide polymorphisms” were included, obtaining 74 results more. After a review of the description of the purpose of these trials on a case-by-case basis and the elimination of duplicates, the final number of clinical trials with PGx-related information amounted to 619. Therefore, only 0.18% of the registered clinical trials contain these terms among the information provided for their inclusion in the system.

Review of the “Start Date” included in the database showed a considerable increase in the number of PGx-related clinical trials, especially from 2000 onwards. Thus, for example, while in 2000 there was only 1 trial with PGx-related information, this number increased to 4 in 2001, to 5 in 2002, to 12 in 2003, to 21 in 2004, to 31 in 2005. The year with the highest number of registered PGx-related clinical trials is 2010, with 53 trials.

With regard to PGx-related clinical trials, the areas of Oncology (28.43%) and Mental Health (10.66%), account for the largest number of trials registered.

**FIGURE 1:**
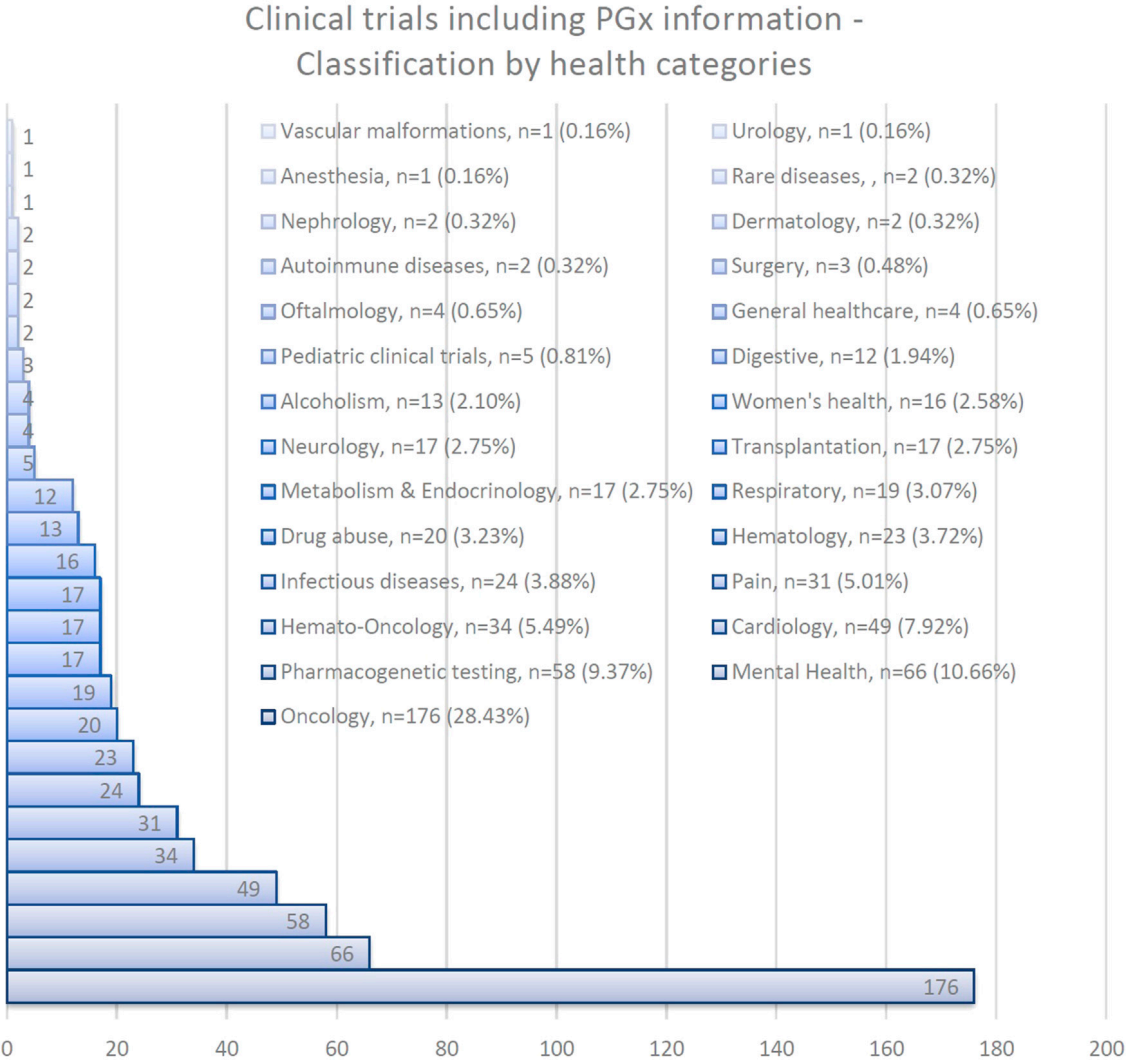
Clinical trials including PGx information classified by health categories.

Figure 1 provides a schematic overview about the clinical trials that include PGx information classified by health categories.

Regarding the recruitment status of these clinical trials, 376 of them are listed on ClinicalTrials.gov as completed (which means the study has ended normally and the last participant’s last visit has been performed); 50 are terminated (the clinical trial has stopped early, will not start again and participants are no longer being examined or treated); 22 were withdrawn (the study stopped before enrolling its first participant); 37 are active, but not recruiting (the study is ongoing, but potential participants are not currently being enrolled); 10 are listed as not yet recruiting; 49 clinical trials are now recruiting; 2 are accessible to enroll by invitation; 2 were suspended; and there is no status information available about 71 of them.

On the reasons for the 2 clinical trials that figure as suspended, one informed that there were difficulties in recruiting patients and the other is waiting for the sponsor to raise funds for the remainder of the study.

In terms of participant characteristics, 542 clinical trials include both male and female participants, 49 trials include only women and 28 are male-only. With respect to subject’s age, there were 42 clinical trials that include both child and adult participants, 546 clinical trials allow adult participants only (participants’ age range has to be equal to or greater than 18 years) and 31 clinical trials aim at paediatric participants (under the age of 18).

For a description on the different clinical trials designs, see Table 1.

**TABLE 1:** Clinical trials including PGx information classified by design.

	Design characteristics	*N* = 619
Clinical Trial Phase	Early phase 1	………………………………………………………………………………………………... 4
	Phase 1	……………………………………………………………………………………………… 105
	Phase 1/Phase 2	………………………………………………………………………………………………. 13
	Phase 2	……………………………………………………………………………………………… 137
	Phase 2/Phase 3	……………………………………………………….……………………………………… 23
	Phase 3	……………………………………………………….……………………………………… 68
	Phase 4	……………………………………………………………………………………………… 127
	Not applicable	……………………………………………………………………………………………… 142
Clinical Trial Allocation	Randomized (*n* = 327)	Crossover assignment: …………………………….……………………………………………… 62
		Factorial assignment: …………………………………………………………………………….. 12
		Parallel assignment: …………………………………………………………………………….. 237
		Sequential assignment: ………..………………………………………………………………….... 2
		Single-group assignment: ……..…………………..…………………………………………….… 12
		No assignment information available: ………………………………………………………………... 2
	Non-Randomized (*n* = 107)	Crossover assignment: …………………………………………………………………………….. 1
		Parallel assignment: ……………………………….……………………………………………... 53
		Single-group assignment: …………………………………………………………………………. 47
		No information available: ………………………………………………………………………….. 6
	Not applicable	………………………………………..…………………………………………………….. 174
	No data	………………………………………………..…….……………………………………..… 11
Clinical Trial Masking	None or Open Label	…………………………………………………….………………………………………... 403
	Single (*n* = 37)	Investigator: ……………………………………..….………………………………………….... 3
		Care provider: ………………………………..…….……………………………………………. 1
		Participant: …………………………………………………………………………………….. 17
		Outcomes assessor: ………………………………..………………………………………….…. 14
		Not described: …………………………………………………………………………………… 2
	Double (*n* = 65)	Participant and Investigator: ………………………………………………………………………. 30
		Participant and Care provider: ……………………………………………………………………… 8
		Care provider and Investigator: ……………………..………………………………………………. 1
		Investigator and Outcomes assessor: ……………….………………………………………………… 6
		Participant and Outcomes assessor: ……………….……………………………………………….... 10
		Not described: ……………………………………..…………………………………………… 10
	Triple (*n* = 28)	Participant, Investigator and Outcomes assessor: ……………………………………………………… 14
		Participant, Care provider and Investigator: …………………………………………………………... 13
		Participant, Care Provider and Outcomes assessor: ..……………………………………………………. 1
	Quadruple (*n* = 71)	Participant, Care provider, Investigator and Outcomes Assessor: …………………………………………... 71
	No data	……………………………………………………………………………….……………… 15

The information available at the ClinicalTrials.gov website was reviewed for each of the results to find out which genes or genetic variants were studied in each clinical trial. This revealed that in many cases this information had not been detailed to the registry. A large number of the studies mentioned the conduct of pharmacogenetic tests in the clinical trials in a broad manner, without specifying further. From the total number of registries, 274 (44.26%) report information indicating which genes or genetic variants are planned to be explored in the clinical trials. See Table 2 for information regarding the results observed on this point.

**TABLE 2:** Most frequently studied genes in clinical trials including PGx information.

Gene	Number of CTs identified	Main health categories
*CYP2D6*	46 CTs	Mental Health, *n* = 13 (28.26%)
		Pain, *n* = 11 (13.91%)
*CYP2C9*	43 CTs	Hematology, *n* = 16 (37.21%)
*CYP2C19*	39 CTs	Cardiology, *n* = 11 (28.21%)
		Mental Health, *n* = 8 (20.51%)
*ABCB1*	39 CTs	Oncology, *n* = 11 (28.21%)
		Transplantation, *n* = 5 (12.82%)
		Cardiology, *n* = 5 (12.82%)
*CYP3A5*	34 CTs	Transplantation, *n* = 9 (26.47%)
		Oncology, *n* = 8 (23.53%)
*CYP3A4*	31 CTs	Oncology, *n* = 10 (32.26%)
		Pain, *n* = 5 (16.13%)
*VKORC1*	27 CTs	Hematology, *n* = 17 (62.96%)
*UGT1A1*	23 CTs	Oncology, *n* = 16 (69.57%)
*COMT*	19 CTs	Mental Health, *n* = 6 (31.58%)
		Pain, *n* = 4 (21.05%)
*CYP2B6*	17 CTs	Infectious diseases, *n* = 4 (23.53%)
*SLCO1B1*	15 CTs	Cardiology, *n* = 5 (33.33%)
*OPRM1*	12 CTs	Pain, *n* = 4 (33.33%)
*DPYD*	11 CTs	Oncology, *n* = 7 (63.64%)
*CYP4F2*	9 CTs	Hematology, *n* = 3 (33.33%)
		Cardiology, *n* = 2 (22.22%)

CTs, stands for clinical trials.

After obtaining this global information, for those health areas with the largest number of PGx -related clinical trials, the primary purpose defined on ClinicalTrials.gov was also consulted.

As shown in Table 3, the clinical trials' primary purpose is focused on treatment.

**TABLE 3:** PGx-related registered clinical trials’ primary purpose.

	Clinical trial primary purpose								
	Basic Science	Health Services Research	Prevention	Screening	Diagnostic	Treatment	Supportive care	Other	No data
Oncology (*n* = 176)	1 (0.57%)	5 (2.84%)	6 (3.41%)	1 (0.57%)	4 (2.27%)	150 (85.23%)	4 (2.27%)	4 (2.27%)	1 (0.57%)
Mental Health (*n* = 66)	5 (7.58%)	2 (3.03%)	0	1 (1.52%)	4 (6.06%)	44 (66.66%)	6 (9.09%)	0	4 (6.06%)
Pharmacogenetic testing (*n* = 58)	9 (15.52%)	6 (10.34%)	2 (3.45%)	4 (6.90%)	4 (6.90%)	18 (31.03%)	4 (6.90%)	4 (6.90%)	7 (12.06%)
Cardiology (*n* = 49)	4 (8.16%)	2 (4.08%)	5 (10.20%)	1 (2.04%)	4 (8.16%)	29 (59.18%)	0	2 (4.08%)	2 (4.08%)
Hemato-oncology (*n* = 34)	1 (2.94%)	0	1 (2.94%)	0	1 (2.94%)	29 (85.29%)	0	2 (5.89%)	0
Pain (*n* = 31)	2 (6.45%)	0	0	1 (3.23%)	2 (6.45%)	22 (70.97%)	2 (6.45%)	2 (6.45%)	0
Infectious diseases (*n* = 24)	1 (4.17%)	1 (4.17%)	1 (4.17%)	0	0	19 (79.17%)	0	1 (4.17%)	1 (4.17%)
Hematology (*n* = 23)	0	0	2 (8.70%)	1 (4.35%)	2 (8.70%)	13 (56.52%)	1 (4.35%)	1 (4.35%)	3 (13.04%)

The countries with the highest number of PGx-related registered clinical trials are United States (*n* = 282, 45.56%), France (*n* = 57, 9.21%), Canada (*n* = 22, 3.55%), Netherlands (*n* = 19, 3.07%), Germany, United Kingdom (*n* = 18, 2.91% each one), Spain (*n* = 17, 2.75%) and the Republic of Korea (*n* = 16, 2.58%). It should be noted that 43 clinical trials (6.95%) are conducted at sites across different countries. There is no location data available for 13 registers (2.10%).

## 4 Discussion

PGx is still in the process of being incorporated into clinical trials. In this work, only 0.18% of the registered clinical trials contained any kind of PGx-related information. In general, the number of clinical trials including PGx information has been reported to be minimal (Burt and Dhillon, 2013).

In relation to the reported geographical distribution, the United States accounted for the greatest number of registered trials, which is consistent with the fact that this is also the region with the highest number of clinical trials overall, according to the data reported at WHO International Clinical Trials Registry Platform (ICTRP) (WHO ICTRP, 2023).

In this search, it was found that the area with the highest number of clinical trials with PGx information was Oncology. Within this therapeutic area, PGx plays a fundamental role, since in certain types of tumours, the knowledge of specific mutations can determine which treatment choice should be made (Filipski et al., 2014). In a review published by Sissung et al., the authors found that despite the large number of published clinical trials in Oncology (over ten-thousand phase I studies), fewer than 1% of these trials referred to the use of PGx in participants’ stratification to optimise the design (Sissung and Figg, 2022).

Another therapeutic area where the incorporation of this type of information is particularly noteworthy is Mental Health. The development of clinical trials that include PGx information may help to improve our understanding about the mechanism of action of drugs used for the treatment of many psychiatric disorders (Pickar and Rubinow, 2001). A growing number of clinical trials with PGx information are appearing in the Mental Health field. As an example, Vos et al. published the results of a randomised clinical trial in patients with depression where the incorporation of pharmacogenetics-informed treatment (PIT) for tricyclic antidepressants was considered. The results showed that PIT allowed for therapeutic concentrations of tricyclic antidepressants to be reached earlier and also resulted in both fewer and less severe adverse effects (Vos et al., 2023). There is a systematic review and meta-analysis published in 2022 focused on examining prospective controlled clinical trials with PGx tests to assess the remission of depressive symptoms. The results of this work suggest a modest but significantly favorable effect of PGx-guided antidepressant therapy on depressive symptom remission (Brown et al., 2022).

Cardiology is another therapeutic area in which the number of reported clinical trials is particularly significant. Nevertheless, the translational of these Cardiology clinical trials’ results into the clinic has been difficult for multiple issues, including the mixed results reported (McDonough, 2021) and also the different evidence support available depending of the specific pharmacological group evaluated (there are trials available regarding antiplatelet therapy, warfarin dosing, statin selection) (Duarte and Cavallari, 2021; Pereira et al., 2020; Claassens et al., 2019; Gage et al., 2018; Notarangelo et al., 2018; Bergmeijer et al., 2014; Voora et al., 2009).

When evaluating the incorporation of PGx from the very beginning of the drug development process, there are some benefits that should be mentioned. One of these benefits is an increased safety of clinical trials (Aneesh et al., 2009), as PGx may help to reduce patients’ exposure to therapies that they have been identified in advance as not being responders to, or that may even be harmful to them (Gerogianni et al., 2018; Su et al., 2016; Plumpton et al., 2016; Franc, 2008; Ingelman-Sundberg, 2008). Another benefit is the reduction in drug development costs (Verbelen et al., 2017; Haycox et al., 2014; Aneesh et al., 2009) as well as in some time-related issues. It has been reported that including PGx in the early phases of clinical trials could contribute to reduce time both for the development process of a new medicine itself, as well as the time for its marketing (Pandya, 2017).

As interest in PM development is growing, it may be worth assessing how PGx information contributes at clinical trials’ performing. This study attempts to provide an insight into pharmacogenetics and PGx’s involvement in registered clinical trials.

Among the limitations of this study, it should be noted that there is not standarization in clinical trials’ databases on how to reflect the type of contribution made by PGx to trials: patients' stratification, pharmacogenetic test to guide dosage, pharmacogenetic test to study safety (adverse drug reactions), PGx-pharmacokinetics association study, therapy response assessment, etc. Therefore, more efforts should be made to propose improvements in this area.

There is currently an on-going guideline proposal, called STROPS (STrengthening the Reporting Of Pharmacogenetic Studies) that has integrated input from researchers, systematic reviewers and journal editors with the aim of improving the completeness and transparency of reports of PGx studies (Chaplin et al., 2020; Richardson et al., 2019; Strops, 2023).

It must also be noted that, besides the potential benefits associated to the incorporation of PGx to clinical trials, it is important to take into account that there is a parallel need to assess potential ethical risks when using genetic information in clinical research. The challenge is to strike a balance between the genetic information required for clinical trials without exposing participants to inappropriate use of their genetic data (Galende-Domínguez and Rivero-Lezcano, 2023). McKinnon et al. stated that ethical issues could be grouped into 3 categories: the equitable provision of healthcare, the possibility that genetic variants may track with race or ethnicity, and the questions of consent, access and privacy surrounding PGx information (McKinnon et al., 2007).

These ethical issues do not differ from those arising in other clinical circumstances (Gershon et al., 2014). For these reasons, it is recommended that genetic testing in clinical trials should be limited to the accomplishment of the main objectives stated in the approved protocol (Galende-Domínguez and Rivero-Lezcano, 2023).

In conclusion, it is certain that PGx should be integrated in clinical trials as a tool that can contribute to a better understanding about drugs efficacy and safety. Nevertheless, researchers and clinicians may not have sufficient PGx training as it has been previously reported (Behr et al., 2023; Kim et al., 2020; Chan et al., 2017), and this is a key point for them to be able to make a proper interpretation of all the PGx’s data. There are now a number of pharmacogenetics and PGx information sources that clinicians and researchers should be familiar with and learn how to use, such as the Clinical Pharmacogenetics Implementation Consortium (CPIC) guidelines (Clinical Pharmacogenetics Implementation Consortium, 2023; Caudle et al., 2014) or the very extensive information available at PharmGKB website.

Some proposals for improving training in this area include specific programmes for health science disciplines in faculties. In this sense, Gurwitz et al. published an article to enhance implementation of PGx and PM into core medical education and practice (Gurwitz et al., 2005). Different proposals have already been suggested by other authors over the last few years (Mosquera and Aleksunes, 2023; Haga et al., 2012; Pulley et al., 2012; Zgheib et al., 2011). Among them, as well as highlighting the importance of including specific programmes for health science disciplines already at the faculty level, it has been noted the importance of developing this knowledge at the residency. Some centres have already tested the implementation of PGx in their clinical practice and there are some publications reflecting the results (Caraballo et al., 2017).

For an assessment of the ethical aspects of PGx studies, there is a report from the Nuffield Council on Bioethics that can be consulted by health professionals and researchers (The Nuffield Council on Bioethics report, 2003; Corrigan, 2005).

All these efforts will contribute for a better drug prescribing and an improvement in patients’ care, which by definition would lead to a PM for providing more individualised treatments.

## Data Availability

All data generated or analyzed during this study are included in this article. Further enquiries can be directed to the corresponding author.
