# Supplementary material for: Pharmacogenomics in clinical trials: an overview
Source: Front Pharmacol. 2023 Oct 20;14:1247088. doi: 10.3389/fphar.2023.1247088 (PMC10625420; doi:10.3389/fphar.2023.1247088)
Supplement: Supplementary file 1 [file DataSheet1.docx]

Supplementary Material

Pharmacogenomics in clinical trials: an overview

Rita Nogueiras-Álvarez^1*^

^1^ Bioaraba Health Research Institute, Clinical Trials Unit, Vitoria-Gasteiz, Araba/Álava, Spain

***** Corresponding Author: [rita.nogueirasalvarez@bioaraba.org](mailto:rita.nogueirasalvarez@bioaraba.org)

# Supplementary Data

The Pharmacogenomics Knowledge Base (PharmGKB) [PharmGKB website^^[[1]](#footnote-1)^^; Whirl-Carrillo et al., 2021; Whirl-Carrillo et al., 2012] is a publicly accessible tool created in 2000 as part of the Pharmacogenomics Global Research Network (PGRN) [PGRN website^^[[2]](#footnote-2)^^] funded by the National Institutes of Health (NIH) [NIH website^^[[3]](#footnote-3)^^]. PharmGKB provides information about the impact of human genetic variation on drug responses. The "Drug Label Annotations" section provides pharmacogenetic information included in the summary of product characteristics of drugs approved by the U.S. Food and Drug Administration (FDA) [FDA website^^[[4]](#footnote-4)^^], the European Medicines Agency (EMA) [EMA website^^[[5]](#footnote-5)^^], the Swiss Agency for Therapeutic Products (Swissmedic) [Swissmedic website^^[[6]](#footnote-6)^^], Health Canada (Santé Canada) (HCSC) [HCSC website^^[[7]](#footnote-7)^^] and the Pharmaceuticals and Medical Devices Agency of Japan (PMDA) [PMDA website^^[[8]](#footnote-8)^^].

**Pharmacogenetic information annotations approved by different regulatory agencies** [source: PharmGKB]

When we search PharmGKB's Drug Label Annotations section [PharmGKB Drug Label Annotations^[[9]](#footnote-9)^], we notice that among the different regulatory agencies included in its register, FDA is the one accounting for the largest amount of pharmacogenetic information on drug products' labeling.

Supplementary Table 1 shows the account of pharmacogenetic information annotations approved by different regulatory agencies [last access on 6 June 2023].

| REGULATORY AGENCIES | NUMBER OF DRUGS WITH AVAILABLE PGX RECOMMENDATION * | "PGx Level" tag count from PharmGKB | | | |
| --- | --- | --- | --- | --- | --- |
|  |  | Testing required | Testing Recommended | Actionable PGx | Informative PGx |
| US Food and Drug Administration (FDA) | 430 | 137 | 7 | 146 | 140 |
| European Medicines Agency (EMA) | 206 | 85 | 5 | 45 | 71 |
| Swiss Agency of Therapeutic Products (Swissmedic) | 130 | 9 | 5 | 92 | 24 |
| Health Canada (Santé Canada) (HCSC) | 182 | 69 | 6 | 67 | 40 |
| Pharmaceuticals and Medical Devices Agency, Japan (PMDA) | 52 | 14 | 1 | 29 | 8 |

*The number of drugs with available pharmacogenetic recommendations per agency do not exactly correspond with the sum of “PGx level” categories, as some drugs may be assigned different tags with different levels of recommendation depending on the indication for which they are used.

The PharmGKB "PGx Level" tags are different levels of action that PharmGKB suggests as interpretation of label contents [PharmGKB PGx Level^[[10]](#footnote-10)^]. The following categories can be considered:

- “Testing required” is assigned when the drug label states or implies that some sort of gene, protein or chromosomal testing should be conducted on the gene or gene product mentioned in the annotation before using a drug.
- “Testing recommended”, when the product information recommends to perform some sort of gene, protein or chromosomal testing before using a drug.
- “Actionable PGx” is allocated to when the label may contain information about changes in efficacy, dosage, metabolism or toxicity due to gene/protein/chromosomal variants or phenotypes (e.g. "poor metabolizers") or the label may mention contraindication of the drug in a particular subset of patients with particular variants/genotypes/phenotypes. This category does not require gene, protein or chromosomal testing.
- “Informative PGx” category is used when the label contains information stating that particular gene/protein/chromosomal variants or metabolizer phenotypes do not affect a drug’s efficacy, dosage, metabolism or toxicity (or it affects but the effect is not “clinically” significant). “Informative PGx” is also assigned to drugs that appear or appeared on the FDA Biomarker List but do not currently meet the requirements to be assigned as “Testing required”, “Testing recommended” or “Actionable PGx”.

# Supplementary Material References

European Medicines Agency (EMA) website. Available at: <https://www.ema.europa.eu/en>

Health Canada (Santé Canada) website. Available at: <https://www.canada.ca/en/health-canada/services/drugs-health-products.html>

National Institutes of Health (NIH) website. Available at: <https://www.nih.gov/>

Pharmaceuticals and Medical Devices Agency of Japan (PMDA) website. Available at: <http://www.pmda.go.jp/english/>

Pharmacogenomics Global Research Network (PGRN) website. Available at: <https://www.pgrn.org/>

Pharmacogenomics Knowledge Base (PharmGKB) website. Available at: <https://www.pharmgkb.org/>

PharmGKB Drug Label Annotations. Available at: https://www.pharmgkb.org/labelAnnotations (05 May 2023, date last accessed)

PharmGKB PGx Level. Available at: <https://www.pharmgkb.org/page/drugLabelLegend#pgx-level>

Swiss Agency for Therapeutic Products website. Available at: <https://www.swissmedic.ch/swissmedic/en/home.html>

U.S. Food and Drug Administration (FDA) website. Available at: https://www.fda.gov/

Whirl-Carrillo M, Huddart R, Gong L, et al. An Evidence-Based Framework for Evaluating Pharmacogenomics Knowledge for Personalized Medicine. Clin Pharmacol Ther. 2021;110(3):563-572. doi:10.1002/cpt.2350

Whirl-Carrillo M, McDonagh EM, Hebert JM, et al. Pharmacogenomics knowledge for personalized medicine. Clin Pharmacol Ther. 2012;92(4):414-417. doi:10.1038/clpt.2012.96

1. Pharmacogenomics Knowledge Base (PharmGKB) website. Available at: <https://www.pharmgkb.org/> [↑](#footnote-ref-1)
2. Pharmacogenomics Global Research Network (PGRN) website. Available at: <https://www.pgrn.org/> [↑](#footnote-ref-2)
3. National Institutes of Health (NIH) website. Available at: <https://www.nih.gov/> [↑](#footnote-ref-3)
4. U.S. Food and Drug Administration (FDA) website. Available at: <https://www.fda.gov/> [↑](#footnote-ref-4)
5. European Medicines Agency (EMA) website. Available at: <https://www.ema.europa.eu/en> [↑](#footnote-ref-5)
6. Swiss Agency for Therapeutic Products website. Available at: <https://www.swissmedic.ch/swissmedic/en/home.html> [↑](#footnote-ref-6)
7. Health Canada (Santé Canada) website. Available at: <https://www.canada.ca/en/health-canada/services/drugs-health-products.html> [↑](#footnote-ref-7)
8. Pharmaceuticals and Medical Devices Agency of Japan (PMDA) website. Available at: <http://www.pmda.go.jp/english/> [↑](#footnote-ref-8)
9. PharmGKB Drug Label Annotations. Available at: <https://www.pharmgkb.org/labelAnnotations> (05 May 2023, date last accessed) [↑](#footnote-ref-9)
10. PharmGKB PGx Level. Available at: <https://www.pharmgkb.org/page/drugLabelLegend#pgx-level> [↑](#footnote-ref-10)
